# Rational design and characterization of cell-selective antimicrobial peptides based on a bioactive peptide from *Crocodylus siamensis* hemoglobin

**DOI:** 10.1038/s41598-023-43274-9

**Published:** 2023-09-26

**Authors:** Sirinthip Sosiangdi, Lapatrada Taemaitree, Anupong Tankrathok, Sakda Daduang, Sophon Boonlue, Sompong Klaynongsruang, Nisachon Jangpromma

**Affiliations:** 1 Protein and Proteomics Research Center for Commercial and Industrial Purposes (ProCCI), Faculty of Science, Khon Kaen University, Khon Kaen, 40002 Thailand; 2 Department of Biochemistry, Faculty of Science, Khon Kaen University, Khon Kaen, 40002 Thailand; 3 Department of Integrated Science, Faculty of Science, Khon Kaen University, Khon Kaen, 40002 Thailand; 4 Department of Biotechnology, Faculty of Agricultural Technology, Kalasin University, Kalasin, 46000 Thailand; 5 Department of Pharmacognosy and Toxicology, Faculty of Pharmaceutical Sciences, Khon Kaen University, Khon Kaen, 40002 Thailand; 6 Department of Microbiology, Faculty of Science, Khon Kaen University, Khon Kaen, 40002 Thailand

**Keywords:** Biochemistry, Biological techniques, Biotechnology, Microbiology

## Abstract

Antimicrobial resistance is a growing health concern. Antimicrobial peptides are a potential solution because they bypass conventional drug resistance mechanisms. Previously, we isolated a peptide from *Crocodylus siamensis* hemoglobin hydrolysate, which has antimicrobial activity and identified the main peptide from this mixture (QL17). The objective of this work was to evaluate and rationally modify QL17 in order to: (1) control its mechanism of action through bacterial membrane disruption; (2) improve its antimicrobial activity; and (3) ensure it has low cytotoxicity against normal eukaryotic cells. QL17 was rationally designed using physicochemical and template-based methods. These new peptide variants were assessed for: (1) their in vitro inhibition of microbial growth, (2) their cytotoxicity against normal cells, (3) their selectivity for microbes, and (4) the mode of action against bacteria using scanning electron microscopy (SEM), transmission electron microscopy (TEM) and confocal microscopy. The results indicate that all designed peptides have more potent antimicrobial efficacy than QL17 and IL15 peptides. However, only the most rationally modified peptides showed strong antimicrobial activity and minimal toxicity against normal cells. In particular, IL15.3 (hydrophobicity of 47% and net charge of + 6) was a potent antimicrobial agent (MIC = 4–12 μg/mL; MBC = 6–25 μg/mL) and displayed excellent selectivity for microbes (*cf.* human cells) via FACS assays. Microscopy confirmed that IL15.3 acts against bacteria by disrupting the cell membrane integrity and penetrating into the membrane. This causes the release of intracellular content into the outer environment leading to the death of bacteria. Moreover, IL15.3 can also interact with DNA suggesting it could have dual mode of action. Overall, a novel variant of QL17 is described that increases antimicrobial activity by over 1000-fold (~ 5 μg/mL MIC) and has minimal cytotoxicity. It may have applications in clinical use to treat and safeguard against bacteria.

## Introduction

Antimicrobial resistance is a global health concern; the poor adherence to drug courses, the misuse of drugs and improper disposal of drugs has resulted in microbes gradually developing resistance to existing drugs^1^. It is therefore vital to identify new drugs, and especially those with novel mechanisms of action^2^. To this end, antimicrobial peptides (AMPs) are promising as: (1) they typically act by disrupting bacterial membranes instead of inhibiting cellular growth (an unconventional mode of action); (2) they are short and are therefore easy to synthesize; (3) they are biomolecules which naturally results in less environmental toxicity compared to conventional small molecule drugs^3–6^. Indeed, several AMPs are now in clinical use including ambicin (nisin), polymixin B and gramicidin S^7,8^. However, their therapeutic use has been limited by their cytotoxicity in mammalian cells or their ability to lyse eukaryotic cells^3,7^.

Designing AMPs is challenging due to the vast chemical space of peptides and the billions of potential combinations they can generate. An interesting approach to this problem has been to identify AMPs from protein hydrolysates of natural origin. For example, peptides from goat whey protein hydrolyzed by alcalase^9^ and peptic hydrolysate from bovine hemoglobin^10^ show broad-spectrum antibacterial activity. In this context, crocodile blood is interesting as it has been used as treatment for human health, a notable example being its use as an artificial blood product^11^.

Our previous studies demonstrated that pepsin hydrolysis of hemoglobin from *Crocodylus siamensis* blood (CHH) over 8 h produced peptides that have antibacterial activity (MIC_50_ values against *Escherichia coli, Staphylococcus aureus, Klebsiella pneumoniae* and *Pseudomonas aeruginosa* were 20, 20, 20 and 10 μg/mL (w/v) respectively). Mass spectroscopy identified the bioactive peptide from 8 h-CHH to be QL17 (QAIIHNEKVQAHGKKVL, + 2 net charge, 41% hydrophobicity)^12^, which conforms to typical AMPs characteristics (10 to 60 amino acid residues, average = 33; + 2 to + 9 net charge, average = + 3.32; ≥ 30% hydrophobic amino acids)^3,13^.

Herein, we evaluate and design variants of QL17 combined with the physicochemical methods in order to identify a single peptide that is potent against the growth of microbes and has low toxicity to normal eukaryotic cells. We also evaluate the mechanism of action against bacteria using microscopy.

## Results and discussion

### Peptide design, characterization, and synthesis

Pepsin hydrolyzed hemoglobin from *Crocodylus siamensis* was reported to exhibit antibacterial, anti-inflammatory and antioxidant properties^12^. The peptide mixture needed to be used at relatively high concentrations to have an antimicrobial effect (≥ 10 μg/mL). From the mixture, the main bioactive peptide was identified to be the 17-mer QAIIHNEKVQAHGKKVL (QL17). Herein, we focus on evaluating this peptide and other rationally modified variants of it in order to develop a peptide with minimal cytotoxicity in mammalian cells, but has high toxicity in microbes.

**Figure 1:**
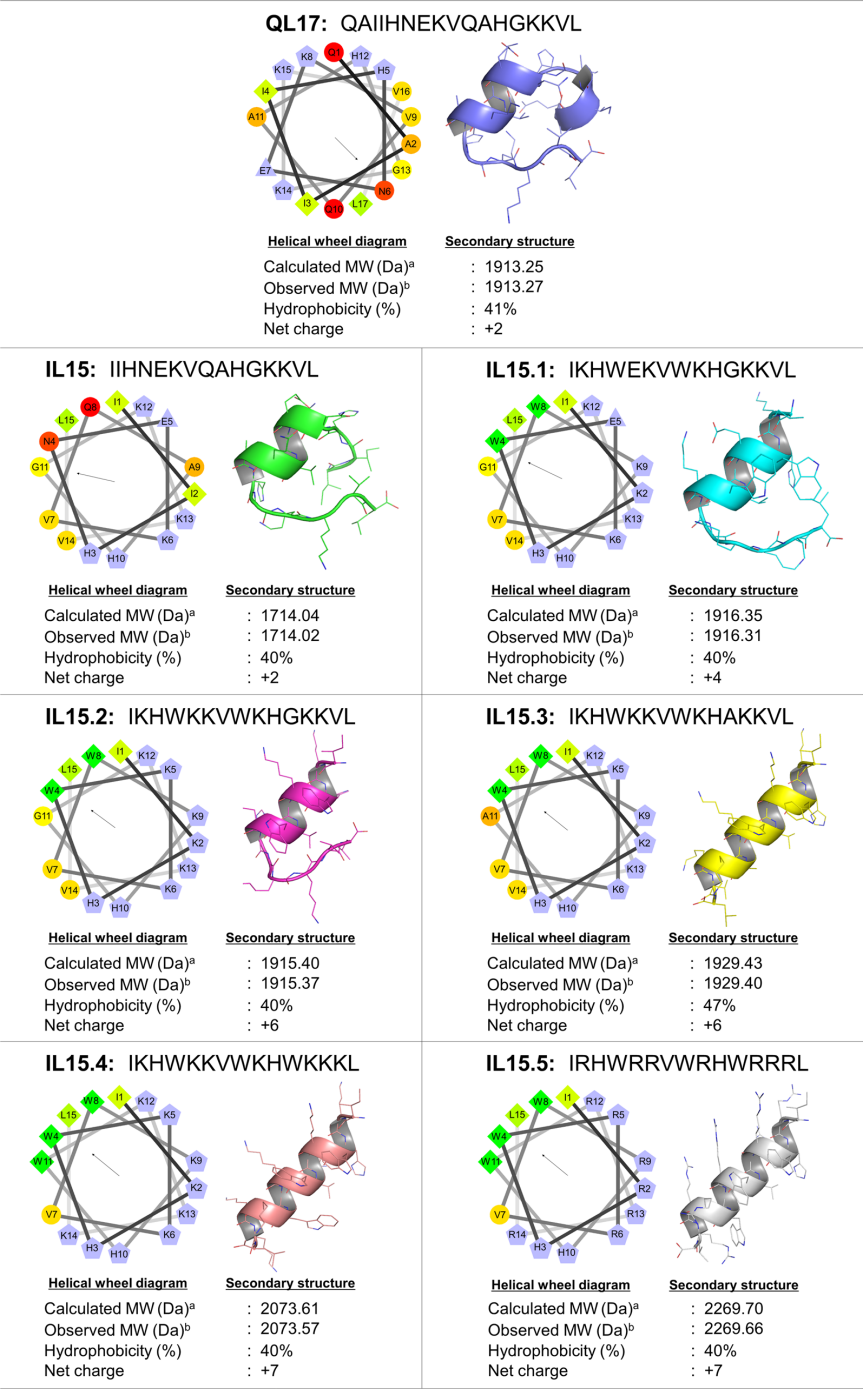
The sequences and physicochemical properties of the newly designed peptides. Helical wheel projection diagrams of the QL17 peptide and its derivatives were constructed. The vector of the hydrophobic moment is shown as an arrow from the center of the helical wheel. The output presents the polar/basic residues in red, polar/acid residues in blue, polar/uncharged residues in green and nonpolar residues in yellow. All peptide secondary structures were built by PEP-FOLD servers and the structure graphic images were created in PyMoL. ^a^Molecular weight (MW) was calculated using the ProtParam tool online at http://web.expasy.org/protparam. ^b^Molecular weight was estimated by mass spectroscopy.

Firstly, Q and A amino acids at the N-terminus of QL17 were removed to yield a 15-mer peptide named IL15 with no significant changes in helical structure, hydrophobicity and net charge. From IL15, four key changes were made to make IL15.1 (I2 → K2; N4 → W4; Q8 → W8; A9 → K9). The I2 and A9 to K substitutions increase the positive charge, whereas A4 and G8 to W substitutions increase hydrophobicity (net + 4 charge, 40% hydrophobicity). More importantly, these changes were made to enhance the amphipathic α-helix of the peptide based on the helical wheel view (Fig. 1); an important property for membranolytic barrel-stave, carpet or toroidal-pore mechanisms that directly disrupt bacterial membrane permeability^3,14^. W residues were chosen to help the peptide adhere to and interrupt the lipid bilayers of the bacterial membrane^15^, while K residues are known to electrostatically interact with the negatively charged microbial lipid membranes and disrupt them^3,5^.

Variant IL15.2 builds on the change of IL15.1 by introducing an E5 → K5 substitution (net + 6 charge, 40% hydrophobicity). IL15.2, therefore, provides a continuous positively charged hydrophilic side of the α-helix. Variant 15.4 builds on IL15.2 with G11 → W11 and V14 → K14 changes (net + 7 charge, 40% hydrophobicity) extending the positively charged hydrophilic side of the α-helix by one residue and enhancing the hydrophobicity of the other side. On the other hand, IL15.3 is a more conservative variant of IL15.2 (G11 → A11, hydrophobicity increases from 40 to 47%). All variants are shown in Fig. 1.

Next, PEP-FOLD was used to predict the secondary structure of the peptides. This showed that all peptides adopt a central α-helix structure. Both QL17 and IL15 have further C- and N-terminal random coils, while IL15.1 and IL15.2 have only a C-terminal random coil. This was encouraging given the α-helical structure is important for membranolytic mechanism by which most AMPs interact with microbes^3,14^.

### Peptide-bacterial membrane interaction model

**Figure 2:**
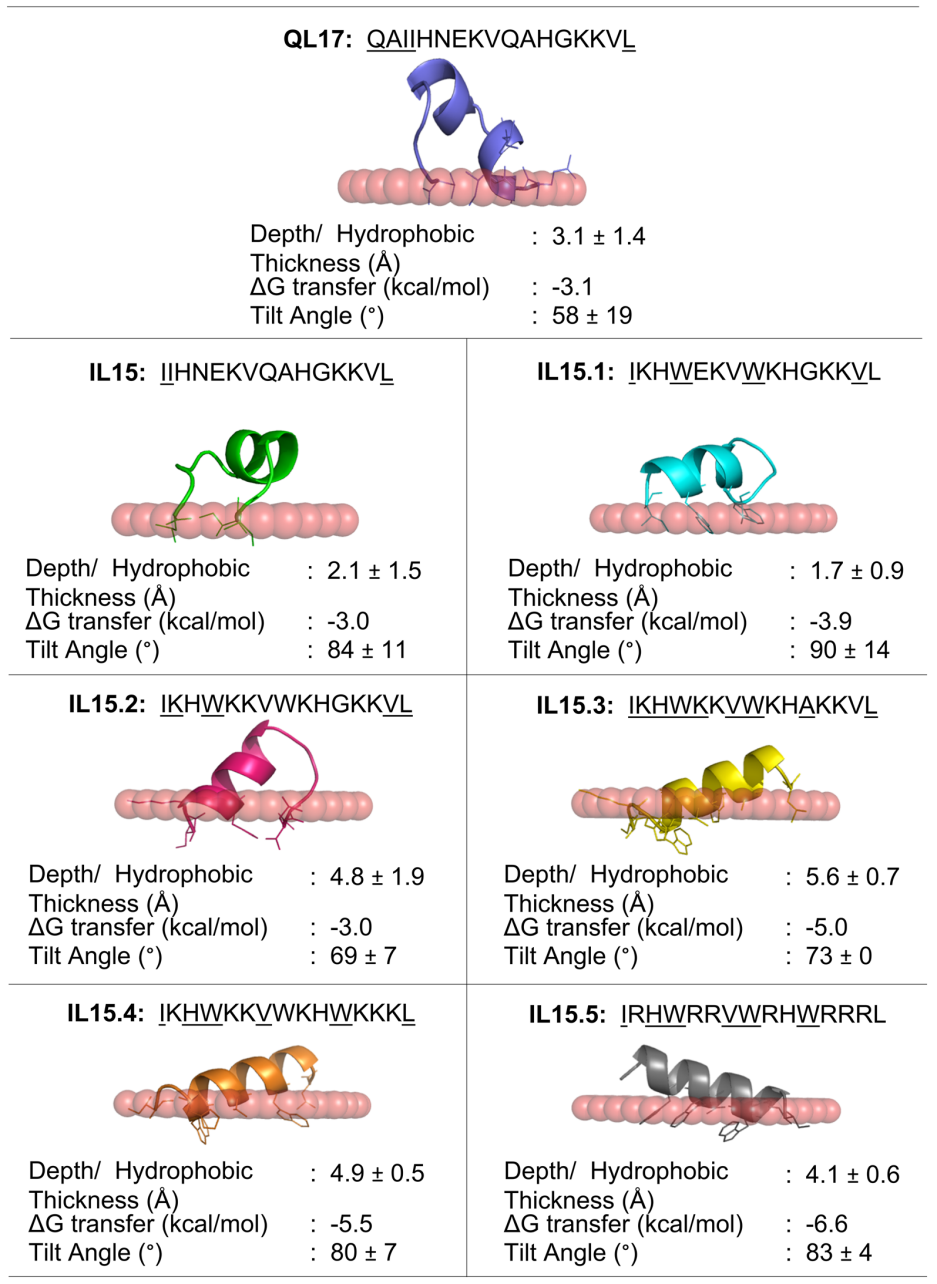
Rational modification of QL17 to increase the depth penetration and amino acid interactions with a membrane. Embedded residues in the peptide sequence are underlined. 3D structures of peptide variants predicted by PEP-FOLD were used to model bacterial membrane interactions using the PPM online server.

The 3D conformation predicted by PEP-FOLD was used in peptide positioning in membrane (PPM) calculation to model the interaction of a peptide with the bacterial membrane (Fig. 2). The results showed the main impact of removing the N-terminal Q and A residues of QL17 to form IL15 was a reduction in tilt angle (58° → 84°) and penetration depth (3.1 ± 1.4 → 2.1 ± 1.5 Å depth), both of which slightly disrupt the membrane. The modifications to enhance the amphipathic α-helix structure in (IL15 → IL15.1) had no major impact on the interaction of the peptide with the membrane despite an increase in the net negative charge (+ 2 →  + 4). This is possibly due to the negatively charged Q residue on the hydrophilic phase of the IL15.1 resulting in a discontinuous positively charged side of the α-helix based on the helical wheel representation of the peptide. Indeed, substituting this Q residue with K (IL15.1 → IL15.2) significantly increased the depth penetration of the peptide (1.7 → 4.8 ± 1.9 Å depth) and tilt angle (90° → 69°). Further enhancing the hydrophobic face of the peptide α-helix (IL15.2 → IL15.3) gave more consistent depth penetration (4.8 ± 1.9 → 5.6 ± 0.7 Å depth). On the other hand, while increasing the positively charged face of the α-helix (IL15.2 → IL15.4, + 4 →  + 6) also made the depth penetration more consistent (4.8 ± 1.9 → 4.9 ± 0.5 Å depth), it came at the cost of a reduced tilt angle (69° → 80°). Finally substituting all K → R (IL15.4 → IL15.5) actually reduced the depth penetration of the peptide (4.9 ± 0.5 → 4.1 ± 0.6 Å depth).

In terms of residues that interact with the membrane, the less charged QL17, IL15.1 and IL15.2 peptides used employed fewer amino acids (only 3–5 residues) to interact with the membrane than the more highly charged variants (IL15.3, IL15.4 and IL15.5, 6–9 residues). This also correlated with the ΔG required for transfer of the peptides from aqueous solutions into the membrane (less charged = − 3.0 to − 3.9 kcal/mol; more charged = − 5.0 to − 6.6 kcal/mol).

The ideal peptide should have high depth penetration and have more residues that interact with the membrane. From our studies, while the original peptide QL17 has reasonable depth penetration, its favourability to transfer into the membrane could be improved (3.1 ± 1.4 Å depth, 58 ± 19° tilt). Rationally designed variant IL15.3 exhibits the best and most consistent predicted membrane interactions (5.6 ± 0.7 nm depth, 73 ± 0° tilt).

### Secondary structure of the peptides by circular dichroism (CD) spectroscopy

**Figure 3:**
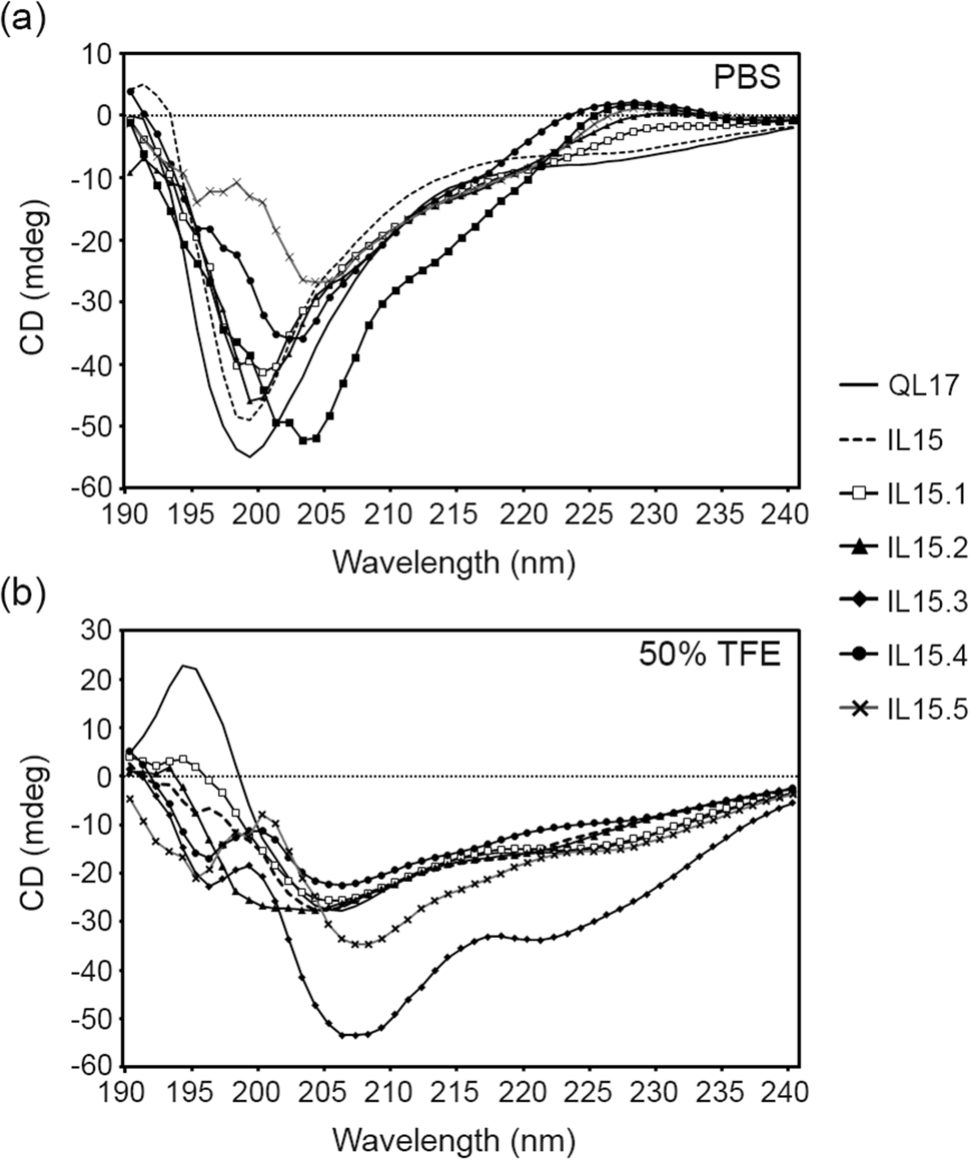
The CD spectra of QL17 variants show (**a**) a random coil structure (minima at 200 and 228 nm) in aqueous PBS solution and (**b**) an α-helical structure (minima at 208 and 222 nm) in 50% TFE/PBS (which mimics a membrane’s local environment). The peptide concentration was 1 μg/mL and CD spectra were recorded from 176 to 240 nm using CD spectroscopy. Data are displayed as mean residue ellipticities.

Using PEP-FOLD servers, all the designed peptides appeared to contain the α-helical conformation (Fig. 1), which is an important characteristic of AMPs^6^. However, the secondary structure of AMPs can vary depending on the local environment (e.g. membrane composition)^16,17^. Therefore, the secondary structure of all peptides was evaluated by CD spectroscopy in PBS (aqueous environment) or 50% trifluoroethanol/PBS (TFE/PBS; mimics the hydrophobic environment of the microbial membrane). In aqueous environments, all peptides displayed a random coil conformation as evaluated by deep and shallow minimums at 200 and 228 nm, respectively (Fig. 3a). In contrast, under a membrane-mimicking (50% TFE) environment, the peptides adopted an α-helical structure, as demonstrated by the two negative peaks at 208 and 222 nm (Fig. 3b). Substitution of many positive residues slightly increased the helicity, which was observed by the lower mean residue ellipticity values at 222 nm. This result agreed with the previous reports that AMPs formed the α-helical structure in the membrane environment, which increases the insertion of the peptide into the membrane and therefore results in increased antimicrobial efficacy^18,19^.

### Antimicrobial activities of peptides

The antimicrobial activity of all peptides was evaluated in vitro using a broth microdilution assay against several microbial strains (Table 1). As predicted by PPM modeling, the peptides could be divided into two groups. Less charged, lower membrane depth penetrating QL17, IL15 and IL15.1 peptides had very high minimum inhibitory concentrations to reduce 90% microbial growth (MIC) (MIC > 200 μg/mL for all strains). On the other hand, more charged, greater membrane depth penetrating peptides were more active. IL15.2 had the most variability in depth penetration and displayed high MIC values against *K. pneumonia*, *P. aeruginosa*, *S. aureus* and *S. epidermidis* (MIC > 200 μg/mL), but some promising results against *E. coli* and *B. subtilis* stain (MIC = 100 and 50 μg/mL respectively). In contrast, the antibacterial activity of IL15.3, IL15.4 and IL15.5, which all gave more consistent membrane penetration, was considerably higher. MIC values were found to range between 4 and 14 μg/mL for IL15.3 and IL15.4 and 8–75 μg/mL for IL15.5, respectively. MBC values were found to be in the range of 6–25 μg/mL for IL15.3, 6–50 μg/mL for IL15.4 and 12–100 μg/mL for IL15.5 respectively.

**Table 1:** MICs (μg/mL) and MBCs (μg/mL) of peptides against several bacterial strains.

Microorganism	QL17		IL15		IL15.1		IL15.2		IL15.3		IL15.4		IL15.5	
	MIC	MBC	MIC	MBC	MIC	MBC	MIC	MBC	MIC	MBC	MIC	MBC	MIC	MBC
*E. coli*	> 200	> 200	> 200	> 200	> 200	> 200	100	150	4	6	4	6	15	25
*K. pneumonia*e	> 200	> 200	> 200	> 200	> 200	> 200	> 200	> 200	10	25	14	50	75	100
*P. aeruginosa*	> 200	> 200	> 200	> 200	> 200	> 200	> 200	> 200	10	18	10	14	35	50
*S. aureus*	> 200	> 200	> 200	> 200	> 200	> 200	> 200	> 200	12	25	12	25	25	50
*B. subtilis*	> 200	> 200	> 200	> 200	> 200	> 200	50	60	8	12	6	10	8	12
*S. epidermidis*	> 200	> 200	> 200	> 200	> 200	> 200	> 200	> 200	9	12	9	12	12	15

As predicted by our computational modeling and rational modifications, IL15.3 exhibited the best antimicrobial activity as predicted by PPM modeling. Interestingly, the potency of IL15.4 (positive charges from K residues) was higher than IL15.5 (positive charges from R residues). This might be due to the deeper insertion of the smaller K side chains (compared to the R side chains)^3^. Moreover, Yang et al.^20^ reported that R-containing peptides showed strong binding affinity to both zwitterionic and anionic liposomes, whereas K-substituted peptides interacted weakly with zwitterionic liposomes but strongly with anionic liposomes.

### Effect of peptides on human erythrocytes and mammalian cells

**Figure 4:**
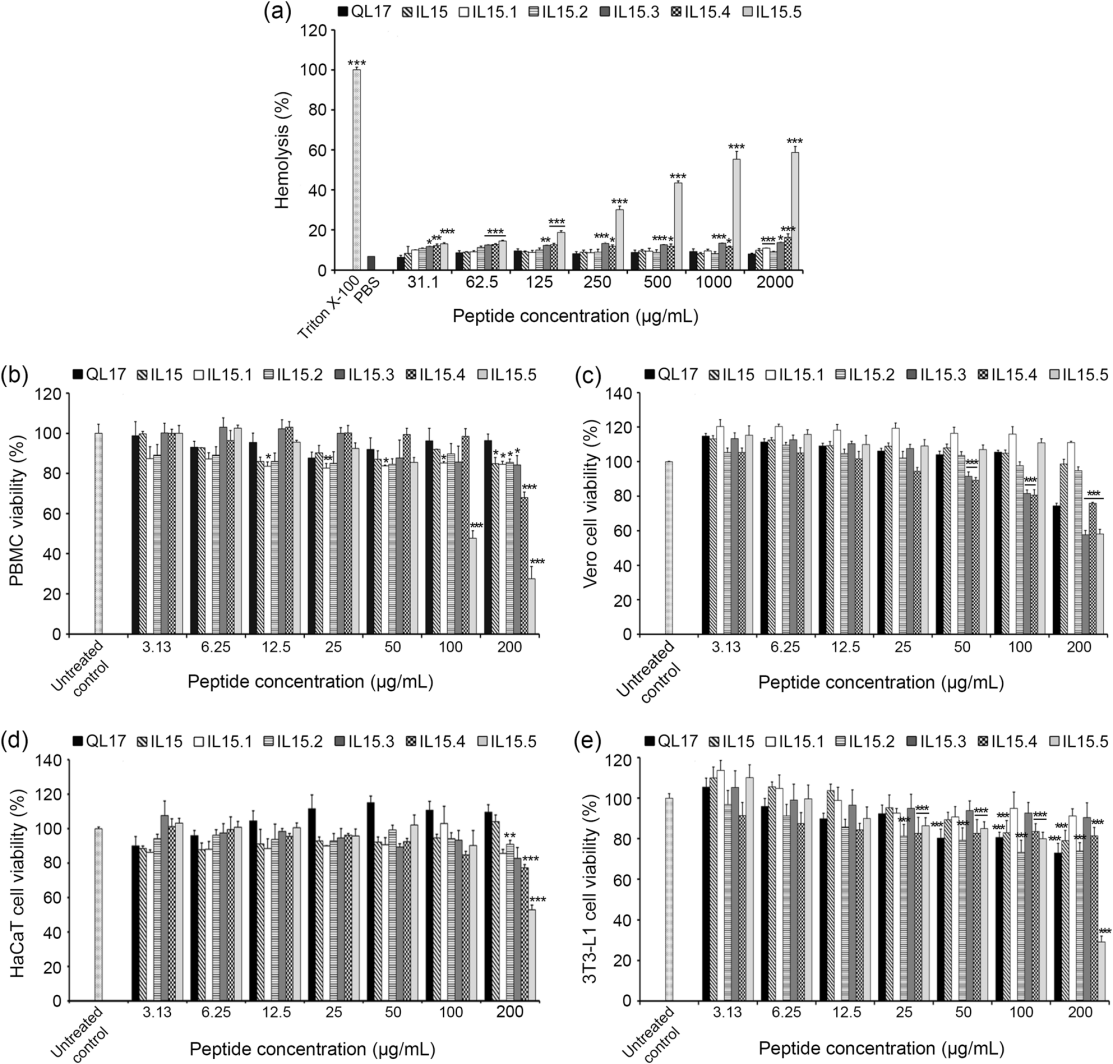
The hemolytic activity against human erythrocytes and cytotoxicity of the peptides on normal cells. (**a**) Human erythrocytes were treated with each peptide at the concentration of 31.3–2000 μg/mL. 1% Triton-X100 was used as the control for 100% hemolysis and the phosphate buffer saline (PBS) treated group was used as a negative control. (**b**), (**c**), (**d**) and (**e**) show the cytotoxicity of the peptides against human peripheral blood mononuclear cell (PBMC), African green monkey kidney cell (Vero), human keratinocyte cell (HaCaT) and mouse preadipocytes (3T3-L1) respectively as measured by the MTT assay. Each value is expressed as the mean ± SD. Asterisks represent statistical significance (*P < 0.05; **P < 0.01; ***P < 0.001) compared with the relevant PBS.

To be an effective therapeutic, the peptides must have low toxicity to normal cells. Therefore, the hemolytic effect and cytotoxicity on normal mammalian cell lines of all peptide variants were examined. Except for IL15.5, all peptides showed insignificant hemolysis at lower concentrations that promote antimicrobial activity and minimal hemolysis at far higher concentrations (25–2000 μg/mL, 8–16%, Fig. 4a). As expected, the only peptide that caused significant hemolysis was IL15.5 (full K → R substitutions relative to IL15.4, HC_50_ = 896 μg/mL, Table S1).

Similar results were found when the cytotoxicity of the peptides was evaluated against the normal cell lines: PBMC (Fig. 4b), Vero cells (Fig. 4c), HaCaT cells (Fig. 4d) and 3T3-L1 cells (Fig. 4e) using the MTT assay. The concentration corresponding to 50% cell death (IC_50_) for PBMC and HaCaT cells was determined from a plot of peptide optical density versus peptide concentration where IC_50_ is the peptide concentration that gives half-maximum optical density (Table S1). Slight cytotoxicity was only observed at the highest concentrations tested (200 μg/mL, ~ 25% reduction in cell viability) for more charged and more membrane penetrating IL15.3, IL15.4 and IL15.5 (Fig. 4b–e). Again, IL15.5 has the highest toxicity toward mammalian cells (IC_50_ = 113 μg/mL for PBMC and IC_50_ = 200 μg/mL for HaCaT; Table S1) confirming R residues are more toxic. An increase in toxicity could be observed when the positive net charge of the peptide was increased possibly due to the less dispersed positive charge of primary amine of K compared to guanidinium group of R residues^3,20^.

**Figure 5:**
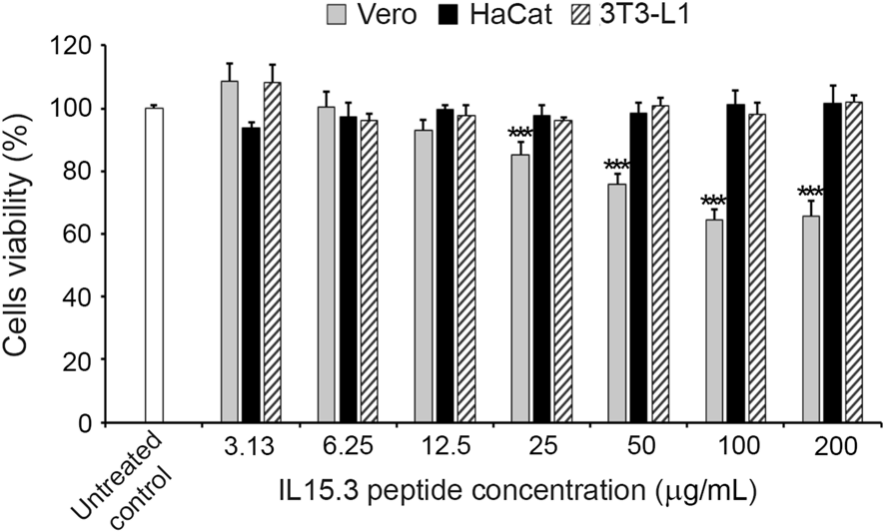
The cytotoxicity of the IL15.3 peptide against African green monkey kidney cell (Vero), human keratinocyte cell (HaCaT) and mouse preadipocytes (3T3-L1) in the absence of fetal bovine serum (FBS) as measured by the MTT assay. Note that MIC and MBC of IL15.3 against bacteria are 4–12 μg/mL and 6–25 μg/mL, respectively. Each value is expressed as the mean ± SD. Asterisks represent statistical significance (***P < 0.001) compared with the untreated control group.

As IL15.3 exhibited the best antimicrobial activity, the cytotoxicity of this peptide on Vero cells, HaCaT cells, and 3T3-L1 cells was evaluated in the absence of fetal bovine serum (FBS) which may sequester the peptides and therefore falsely suggest they have low cytotoxicity. The assay corroborated the previous FBS-containing assay and suggested there is low cytotoxicity with the exception of Vero cells at high concentrations (~ 34% reduction in cell viability at 200 μg/mL; Fig. 5). These results confirmed that IL15.3 at the MIC and MBC cause no cytotoxicity to eukaryotic cells.

### Selectivity index

The selectivity index (SI) is often used to represent the cell selectivity of an antimicrobial therapeutic agent^14^. The SI of the peptides was calculated as the ratio of the 50% hemolysis (HC_50_) or 50% cytotoxicity in a normal cell line (IC_50_) to the MIC of each peptide. The higher SI value indicated the greater selectivity of the peptide towards bacterial membranes over mammalian cell membranes. Considering HC_50_ values, the highest tested concentration of 2000 μg/mL was used for SI calculation of QL17, IL15, IL15.1, IL15.2, IL15.3 and IL15.4, while the concentration of 896 μg/mL (HC_50_) was used for SI calculation of IL15.5. Even for normal cells, the SI was calculated by using the highest tested concentration of 200 μg/mL, except for the IL15.5 treated PBMC and HaCaT cells (Table S2 and Table S3). The results indicated that IL15.3 exhibited the highest cell selectivity. The SI of IL15.4 towards gram-negative and gram-positive bacteria was increased approximately 6 and 25-fold respectively in comparison with IL15.2 (Table S2 and S3). The SI of IL15.4 towards gram-negative and gram-positive bacteria was observed to be slightly lower than that of IL15.3. There is a threshold of an increase in a positive net charge of the peptide for the elevation of antimicrobial activity. For the hydrophobicity of the peptide, IL15.3 (+ 47 hydrophobicity) exhibited a higher SI value than IL15.2 (+ 40 hydrophobicity), suggesting the hydrophobicity could enhance the cell selectivity of peptides. These findings suggested that the substitution of positive net charged and hydrophobic residues in a suitable place will enhance the SI of peptides^14,21^. Previously, Yin et al.^17^ demonstrated that the cell selectivity of the antimicrobial peptide does not rely on only one factor but a balance of (i) helicity, (ii) core segment hydrophobicity, (iii) positive charge distribution, (iv) ability to dimerize/oligomerize in the membrane and (v) low aggregation are required.

### Time kinetics of killing bacteria

**Figure 6:**
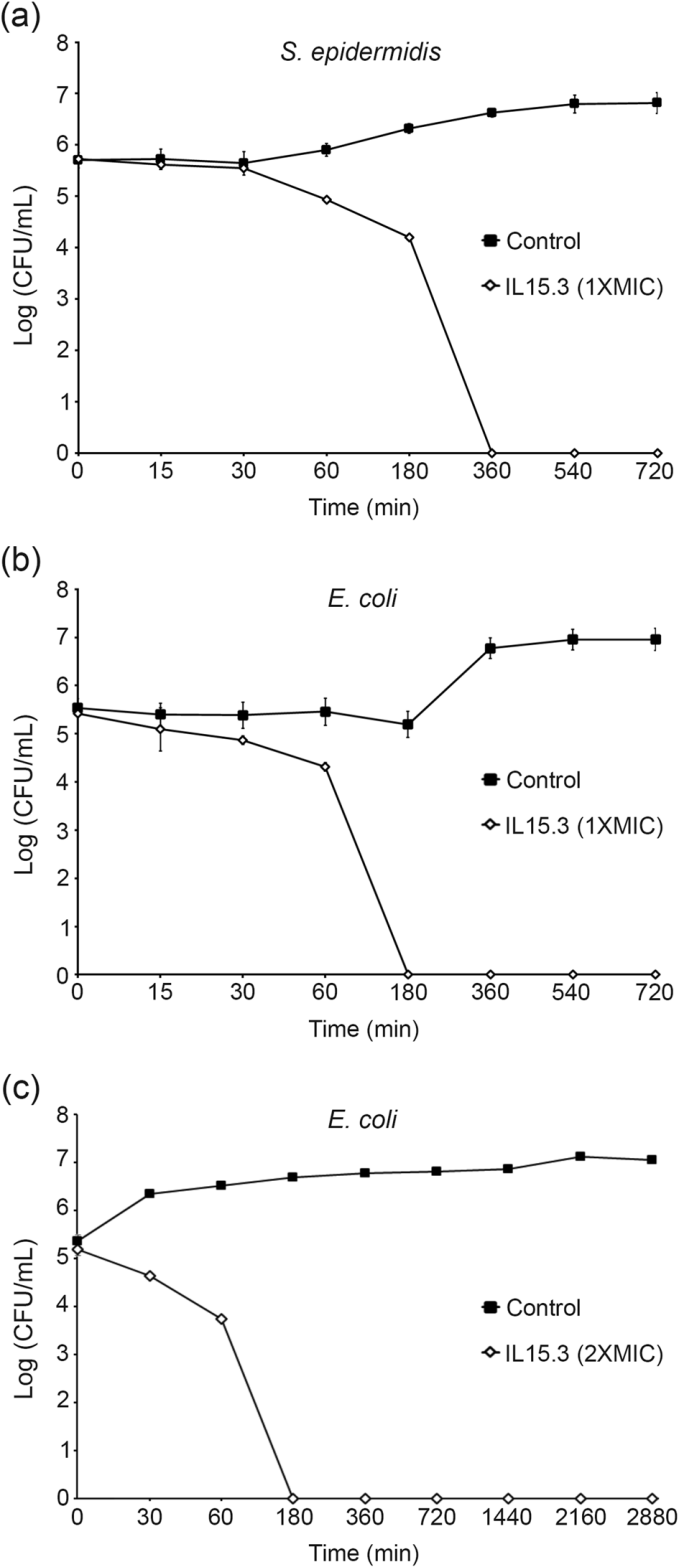
IL15.3 inhibits cell growth after 30–60 min and causes complete cell death by 180–2880 min. (**a**) is the time-killing study of *S. epidermidis* (gram-positive) and (**b**) is the time-killing study of *E. coli* (gram-negative) treated with IL15.3 at 1 × MIC concentration for 0, 15, 30, 60, 180, 360, 540 and 720 min. (**c**) is the time-killing study of *E. coli* (gram-negative) treated with IL15.3 at 2 × MIC concentration for 0, 30, 60, 180, 360, 720, 1440, 2160 and 2880 min.

Of the various peptides tested, IL15.3 was the most promising AMP. As a consequence, subsequent studies focused on a more detailed evaluation of this peptide. First, the kinetics of microbial growth inhibition were tested for *S. epidermidis* and *E. coli* after exposure to 1 × MIC concentration using a time-killing assay. This is an important assay to explore the antimicrobial peptide’s performance^22^. The results showed that IL15.3 was able to inhibit the growth kinetic rate of *S. epidermidis* and *E. coli* within 180 min and 60 min respectively, and completely stop its growth after 360 min and 180 min (Fig. 6a and b). This demonstrates IL15.3 acts more effectively against gram-negative bacteria than gram-positive bacteria and that the bacterial membrane composition is important.

As IL15.3 required more than 30 min to kill bacteria at 1 × MIC, the time-killing assays of *E. coli* using 2 × MIC concentration were performed over longer times to demonstrate that (1) cell death occurs at shorter times, which is supportive of membrane disruption, and (2) the *E. coli* do not adapt and gain resistance to the peptide. As shown in Fig. 6c, the results show IL15.3 began to inhibit growth kinetic rate within 30 min, fully killed the cells within 180 min and did not allow the cells to regrow after 48 h.

### Peptide selectivity using flow cytometry technique

**Figure 7:**
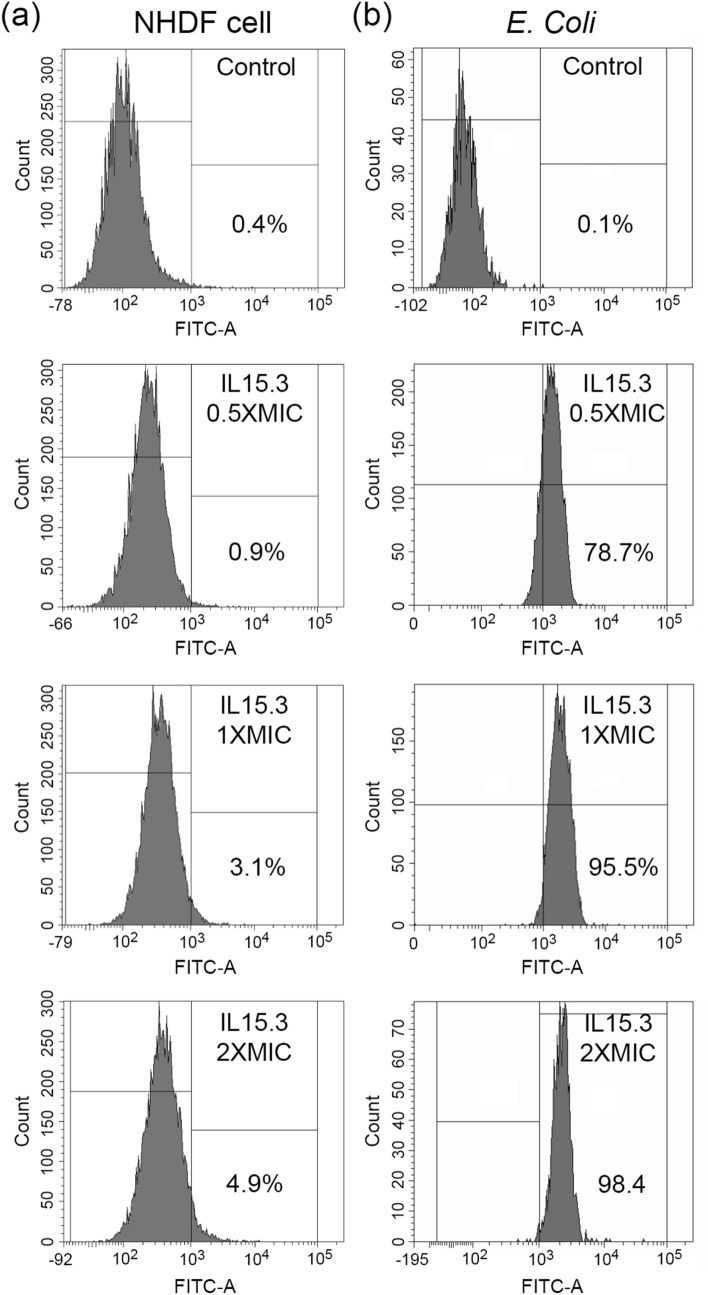
The cell selectivity of FITC-labelled IL15.3 to (**a**) NHDF cells and (**b**) *E. coli* bacteria (gram-negative) as determined by flow cytometry. The control groups in both mammalian and bacterial cells were processed without treatment with FITC-labelled IL15.3. The treatment groups were co-incubated with 0.5 × MIC, 1 × MIC and 2 × MIC of FITC-labelled peptide.

Next the cell selectivity of FITC-labelled IL15.3 for human (NHDF) or bacterial (*E. coli*) cells was evaluated using flow cytometry. In the absence of the peptide, NHDF and *E. coli* cells had no significant fluorescence above the 10^3^ FITC signal threshold (Fig. 7). Similarly, upon treatment of human cells with FITC-labelled IL15.3, only 0.9 to 4.9% of the FITC signal crossed this threshold (Fig. 7a), showing poor uptake by these cells. This corroborates the MTT cytotoxicity assays, which also showed IL15.3 has minimal toxicity to human cells. On the other hand, treatment of *E. coli* cells with FITC-labelled IL15.3 showed significantly more signal above the FITC threshold (78.7 to 98.4% for *E. coli,* Fig. 7b) suggesting the peptide interacts with the cells in some form. These results clearly show that IL15.3 has a very strong preference for interacting with microbes compared to human cells which is critical for therapeutic applications.

### Mode of action of IL15.3

**Figure 8:**
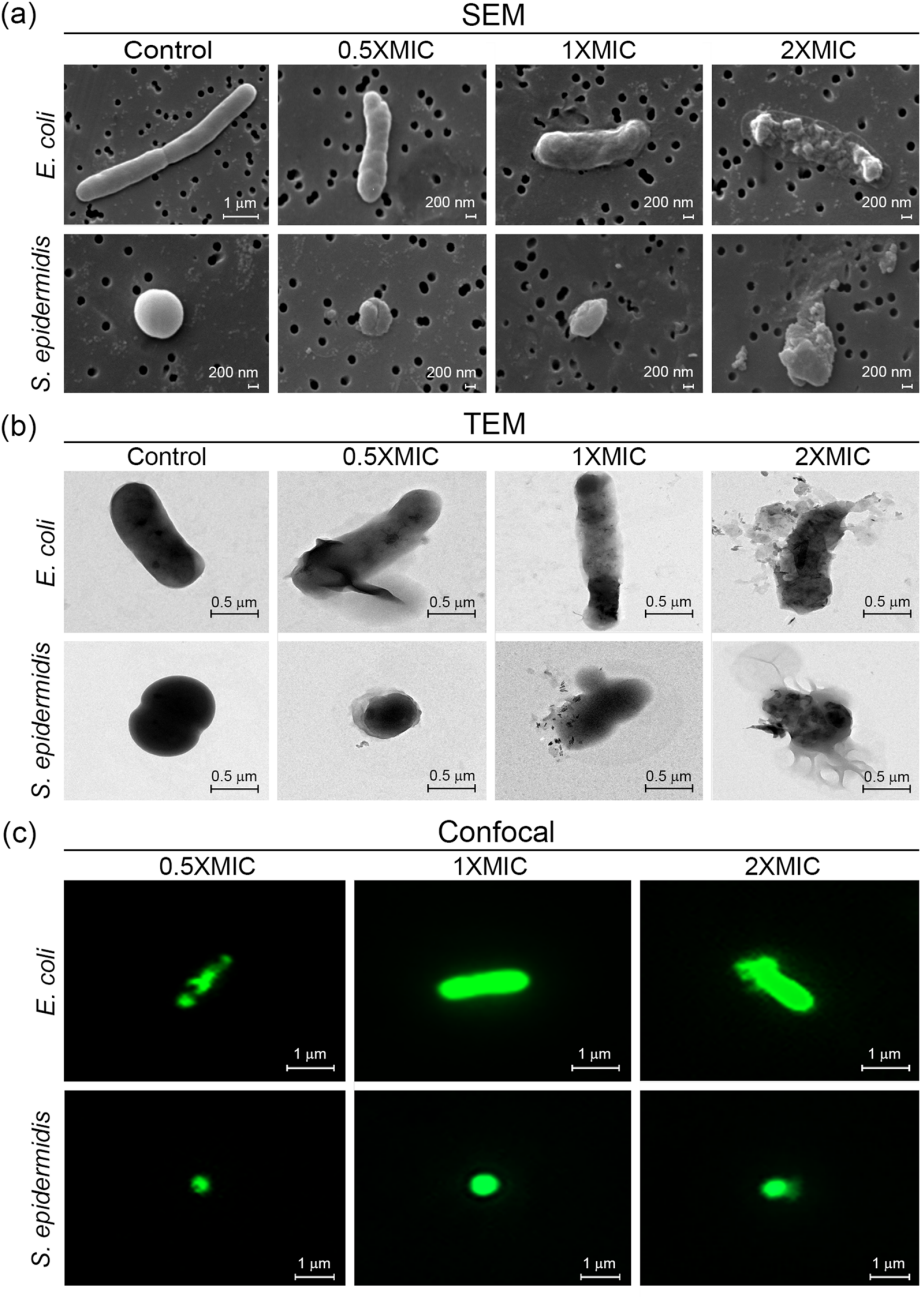
(**a**) SEM, (**b**) TEM and (**c**) confocal microscopy images of *E. coli* and *S. epidermidis* show progressively more severe membrane disruption upon exposure to higher concentrations of IL15.3 (0.5 × MIC, 1 × MIC and 2 × MIC) for 60 min.

Finally, α-helical peptides often cause membranolysis through barrel-stave, carpet or toroidal-pore mechanisms that directly disrupt bacterial membrane permeability^3,14^. As a result, the mode of action of IL15.3 was explored by treating *E. coli* and *S. epidermidis* cells with IL15.3 for 60 min (a time that causes growth inhibition from kinetic assays) and observing them by microscopy. Consistent results were observed by both SEM and TEM for both *E. coli* and *S. epidermidis* cells (Fig. 8a and b). Namely, the cells display a smooth morphology in the absence of the peptide that becomes damaged upon treatment with IL15.3 in a dose-dependent manner. Low concentrations (0.5 × MIC) cause some shrinking of the cells and rough aberrations on the cell membrane. For *E. coli*, cell clumping was also observed. Higher concentrations (1 × MIC) cause even greater shrinkage of the cells as well as some cell membrane rupture. The highest concentrations (2 × MIC) cause a complete loss of membrane integrity with the release of intracellular content. These progressive changes in cell morphology suggest that IL15.3 causes bacteriostasis or bactericide via disruption of the cell membrane.

To cross validate these results, IL15.3 was labelled with FITC and its uptake by *E. coli* and *S. epidermidis* was visualized their localization by confocal microscopy (Fig. 8c). As expected, at lower concentrations (0.5 × MIC) IL15.3 interacted with the surface of both bacterial cells and did not fully penetrate into bacterial cells as indicated by the non-uniform pattern of fluorescence. At higher concentrations (1 × MIC and 2 × MIC), IL15.3 penetrated into bacterial cells as observed by the filled green rod-shaped *E. coli* and filled green coccus-shaped *S. epidermidis*.

### Gel retardation

**Figure 9:**
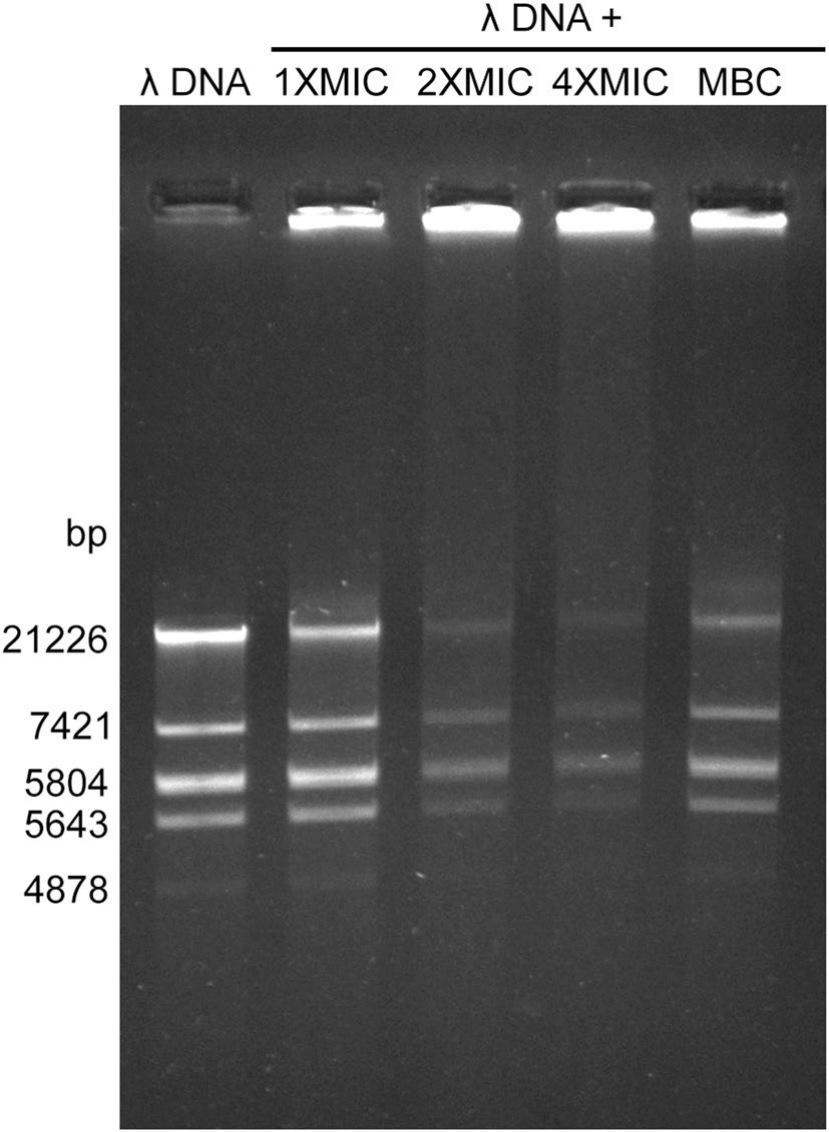
Concentration-dependent binding of IL15.3 peptide with DNA as shown by a gel retardation assay and smearing of the DNA bands at higher concentrations of the peptide. *EcoRI*-digested Lambda (λ) DNA (200 ng) was incubated with different concentrations of IL15.3 at 1 × MIC (4 µg/mL), 2 × MIC (8 µg/mL), 4 × MIC (16 µg/mL) and MBC (6 µg/mL) of *E. coli*. The results were analyzed using 0.8% agarose gel electrophoresis.

In order to investigate the interaction of IL15.3 peptide with bacterial DNA, *EcoRI*-digested Lambda (λ) DNA was incubated for 1 h with or without IL15.3 at concentrations of 1 × MIC, 2 × MIC, 4 × MIC and MBC of *E. coli*, and separated by agarose gel electrophoresis. In the absence of the peptide, the DNA fragment bands were sharp and show no smearing. However, in the presence of the peptide, more DNA remained in the well and the intensity of the lower molecular weight bands progressively became weaker suggesting IL15.3 binds to λ DNA fragments (Fig. 9 and Fig. S1). Several studies demonstrated that cationic antimicrobial peptides such as Buforin II can cross the cytoplasmic membrane and enter the cytoplasm to interact with intracellular DNA^23^. It was speculated that the interaction of antimicrobial peptides with DNA could disrupt gene duplication, transcription and expression, which is an effective way to block protein synthesis and disrupt the life cycle of bacteria^24^. Our results suggest this could also be a possible mechanism of action for IL15.3 given that at lower concentrations (1 × MIC) it takes ~ 1 h to kill microbes and that only higher concentrations (2 × MIC) cause cell death within 30 min (which is more consistent with direct cell membrane disruption).

## Materials and methods

### Design of antimicrobial peptides

Antibacterial peptide QL17 (QAIIHNEKVQAHGKKVL) from *Crocodylus siamensis* hemoglobin hydrolysate was selected as a template for designing new peptides. QL17 has a net positive charge (+ 2) at pH 7 and has good aqueous solubility (41% hydrophobicity). Our modifications focused on introducing lysine (K) or arginine (R) amino acids to increase the net positive charge and varying the hydrophobic residues such as alanine (A), leucine (L), isoleucine (I), phenylalanine (F) or tryptophan (W) to alter the peptide structure. Our objective was to create peptides with a net + 4 to + 7 positive charge with 40 to 47% of hydrophobicity. The 2D structures of QL17 and its derivatives were constructed and the helical wheel projection was constructed online using the Emboss Pepwheel program^25^.

### Peptide solid-phase synthesis

Fmoc solid phase methodology (GL Biochem, Shanghai, China) was used to synthesize all peptides. These peptides were purified by reversed phase high-performance liquid chromatography (≥ 95% purity) and their identity was confirmed using electrospray ionization-mass spectrometry.

### Peptide-membrane interaction modeling

The secondary structure of each peptide was predicted using PEP-FOLD^26^, with the resulting PDB file used to calculate peptide-membrane interactions using PPM (http://opm.phar.umich.edu/server.php)^27^. PyMoL was used to create 3D rendered images of the peptides.

### Peptide circular dichroism

Each peptide was dissolved in 1 X phosphate buffer saline solution (PBS) or 50% trifluoroethanol (TFE) to reach a target concentration of 0.1 μg/mL. CD spectra were recorded at a scanning speed of 20 nm/min at wavelengths from 176 to 240 nm using a circular dichroism spectrometer (Jasco, Easton, MD, USA). Data were displayed as mean residue ellipticities.

### Antimicrobial activity assay

Broth microdilution assays were performed as reported previously^3^. In short, the bacteria including *Escherichia coli* (ATCC 25922), *Klebsiella pneumoniae* (ATCC 27736), *Pseudomonas aeruginosa* (ATCC 27853), *Bacillus subtilis* (ATCC 6633), *Staphylococcus aureus* (ATCC 25923) and *Staphylococcus epidermidis* (ATCC 12228) were grown in Nutrient broth to mid-log phase at 37 °C. The bacterial suspension (50 μL, concentration of 1 × 10^6^ CFU/mL) was loaded into a well with serially diluted peptide (50 µL, final concentrations ranging from 0–200 μg/mL) in a sterile 96-well plate and incubated at 37 °C for 18 h. The optical density (OD) at 600 nm was measured using a microplate reader (ALLSHENG, Zhejiang, China) in order to determine the growth of the bacteria. The inhibition was estimated using the formula;

$$\% \;{\text{inhibition}} = \left( {\frac{{{\text{OD}}_{{600}} \;{\text{of}}\;{\text{control}} - {\text{OD}}_{{600}} \;{\text{of}}\;{\text{sample}}}}{{{\text{OD}}_{{600}} \;{\text{of}}\;{\text{control}} - {\text{OD}}_{{600}} \;{\text{of}}\;{\text{blank}}}}} \right) \times 100$$

where minimal inhibitory concentration (MIC) is defined as the minimum concentration that inhibited 90% of bacterial growth.

In order to evaluate the minimal bactericidal concentration (MBC)—the lowest concentration that terminates the growth of bacteria on an agar plate—of the peptide, co-culture medium (100 μL) from each well after the microdilution assay was spread on a nutrient agar plate. Plates were maintained at 37 °C for 24 h and the number of colonies formed was counted to evaluate antimicrobial activity.

### Erythrocyte hemolysis assay

Human whole blood was obtained from Blood Transfusion Center Faculty of Medicine Khon Kaen University (ethical approval number HE601010). Fresh human erythrocytes were carefully mixed with PBS (1:3) and centrifuged at 1000 × *g* for 2 min. The supernatant was discarded and the cell pellet was resuspended to 2% w/v in PBS. 100 µL of the resuspended erythrocytes were incubated with the peptide (10 µL, final concentrations ranging from 12.5 to 100 μg/mL, two-fold serial dilution) at 37 °C for 1 h. After the treatment, the plate was centrifuged and the supernatant was taken to determine the optical density at 415 nm using a microplate reader (ALLSHENG, Zhejiang, China). The positive control for this assay was the addition of Triton X-100 (1% v/v), which causes 100% hemolysis. The negative control was cells treated with PBS buffer only. The hemolysis activity was estimated using the equation:

$$\% \;{\text{hemolysis}} = \left( {\frac{{{\text{OD}}_{415} \;{\text{of}}\;{\text{sample}}}}{{{\text{OD}}_{415} \;{\text{of}}\;{\text{Triton}}\;{\text{X-}}100\;{\text{treated}}\;{\text{control}}}}} \right) \times 100$$

### MTT cytotoxicity assay

Human peripheral blood mononuclear cells (PBMC), African green monkey kidney cells (Vero), human keratinocyte cells (HaCaT) and mouse preadipocytes (3T3-L1) were used to evaluate the cytotoxicity of peptides by the MTT assay^28^. PBMCs were isolated using Ficoll medium (GE Healthcare, Uppsala, Sweden) as described by Jangpromma et al.^28^. The PBMCs were then cultured in RPMI 1640, while Vero, HaCaT and 3T3-L1 cells were cultured in DMEM. All culture mediums were supplemented with 10% heat-inactivated fetal bovine serum (FBS) and 1% antibiotic:antimycotic (Gibco, USA). The cells were cultured at 37 °C with 5% CO_2_/95% relative humidity. After that, PBMC (2 × 10^6^ cells/well), Vero (1 × 10^4^ cells/well), HaCaT (1 × 10^4^ cells/well) and 3T3-L1 (1 × 10^4^ cells/well) were individually seeded into 96-well plate and cultured at 37 °C overnight. Next, two-fold serially diluted peptides (3.13–200 μg/mL) were added to the cell culture. After incubation in 5% (v/v) CO_2_ incubator for 24 h, the medium culture was removed and MTT solution (0.05 μg/mL final concentration in fresh medium culture) was added to the cells and incubated for 30 min. Thereafter, the medium was removed and DMSO (100 µL) was added to each well to dissolve the formazan crystals. The optical density at 570 nm was determined using a microplate reader (ALLSHENG, Zhejiang, China). The cytotoxicity of IL15.3 against Vero, HaCaT and 3T3-L1 cells in the absence of FBS was also measured by the MTT assay to confirm the peptide is not sequestered by proteins in the serum causing artificially low cytotoxicity against eukaryotic cells.

Cell viability was determined using the equation:

$$\% \;{\text{cell}}\;{\text{viability}} = \left( {\frac{{{\text{OD}}_{570} \;{\text{of}}\;{\text{sample}}}}{{{\text{OD}}_{570} \;{\text{of}}\;{\text{untreated}}\;{\text{control}}}}} \right) \times 100$$

GraphPad Prism8 (San Diego, CA, USA) was used to calculate IC_50_ values using a non-linear regression model.

### Selectivity index

Selectivity index (SI) were calculated from the ratio of the peptide concentrations that caused 50% lysis of erythrocyte (HC_50_) to the minimum inhibitory concentration (MIC), or by the ratio of the peptide concentrations that caused 50% cell death of normal cell death (IC_50_) to the minimum inhibitory concentration (MIC). The formula used was SI = HC_50_ / MIC or SI = IC_50_/MIC.^29^.

### Time-killing kinetics assay

The time-killing kinetics of peptide was determined as described previously by Yaraksa et al.^16^ In short, *E. coli* (ATCC25922) and *S. epidermidis* (ATCC12228) cells grown overnight were diluted in the Nutrient broth to a concentration of 1 × 10^6^ CFU/mL. The diluted bacterial suspension (50 µL) was incubated with the peptide solution (50 µL, final concentration = 1 × MIC or 2 × MIC) at 37 °C. 10 µL of the mixture was taken at regular time intervals (0, 15, 30, 60, 180, 360, 540 and 720 min for 1 × MIC and 0, 30, 60, 180, 360, 720, 1440, 2160 and 2880 min for 2 × MIC), diluted and cultured on nutrient agar plates for 24 h. Bacterial cells were then counted and a graph of the log CFU/mL against incubation time was plotted to evaluate the rate at which peptides kill bacterial cells.

### Cell selectivity

The cell selectivity of FITC-labelled peptides was determined on human fibroblast (NHDF) and *E. coli* using flow cytometry. Briefly, 2 × 10^5^ cells/mL of NHDF cells in DMEM medium supplemented with 10% heat-inactivated FBS and 1% antibiotic:antimycotic (Gibco, USA) were seeded on a 12-well plate and incubated at 37 °C overnight, while *E. coli* were cultured in Nutrient broth at 37 °C until reaching the log phase. Next, both fibroblast and bacterial cells were co-incubated with 0.5 × MIC, 1 × MIC and 2 × MIC of the FITC-labelled peptides in parallel. 1 h later, NHDF cells were harvested by trypsinization. All fibroblast and bacterial cells were centrifuged at 12,000 × *g* for 10 min and the cell pellets were washed with PBS buffer. After PBS buffer was removed, cells were then resuspended in Annexin binding buffer and measured using a flow cytometer (BD Biosciences, San Jose, CA, USA).

### Scanning electron microscopy analysis

*E. coli* and *S. epidermidis* cells were cultured to the log phase at 37 °C before treatment with peptides at a concentration of 0.5 × MIC, 1 × MIC and 2 × MIC at 37 °C for 1 h. Next, the cell pellets were pipetted onto a 0.2 µm polycarbonate membrane and the cells were left there for 10 min. Subsequently, 2.5% (v/v) glutaraldehyde was added for the fixation of cells. After 1 h incubation, bacterial cells were then dehydrated with 30%, 50%, 70%, 90% and 100% aqueous ethanol solutions. The dehydration step took 15 min for each aqueous ethanol concentration. The dry bacterial specimen was placed on a stub with carbon tape. Finally, the stub was overlaid using gold palladium and photographed by SEM (LEO Electron Microscopy Inc., Thornwood, NY).

### Transmission electron microscopy analysis

*E. coli* and *S. epidermidis* cells were cultured to the log phase at 37 °C before treatment with peptides at a concentration of 0.5 × MIC, 1 × MIC and 2 × MIC at 37 °C for 1 h. The cell pellets were fixed with 5% (v/v) glutaraldehyde and 10% formaldehyde for 10 min. Bacterial cells were loaded on a copper grid at each interval, rinsed with distilled water and dried in a desiccator. The image was then visualized using TEM (FEI Company, Hillsboro, OR, USA).

### Confocal microscopy analysis

*E. coli* and *S. epidermidis* cells were cultured to the log phase at 37 °C and harvested by centrifugation. The cells were washed 3 times with PBS buffer, resuspended in PBS buffer, and then diluted to a final optical density of 0.001 at 600 nm. Subsequently, the cells were treated with the FITC-labelled IL15.3 peptide at concentrations of 0.5 × MIC, 1 × MIC and 2 × MIC. After incubation for 1 h, the treated bacterial cells were centrifuged at 5000 rpm for 15 min. The pellet was collected, washed 3 times with PBS and then resuspended in PBS. The location of FITC-labelled IL15.3 was then visualized by a confocal microscope (Thermo Fisher Scientific, Waltham, MA, USA) with an excitation wavelength of 485 nm and an emission wavelength of 521 nm.

### Gel retardation assay

DNA used for this test was Lambda (λ) DNA, which had been digested with *EcoR*I into fragments sized 21,226, 7421, 5804, 5643, 4878 and 3530 bp (Thermo Fisher Scientific, Waltham, MA, USA). The gel retardation assay was run by mixing 200 ng of λ DNA with different concentrations of IL15.3 at 1 × MIC (4 µg/mL), 2 × MIC (8 µg/mL), 4 × MIC (16 µg/mL) and MBC (6 µg/mL) of *E. coli*. The mixtures were incubated for 60 min at room temperature and subjected to electrophoresis on a 0.8% agarose gel. Gel was then stained with ViSafe Red gel stain (Vivantis, Selangor Darul Ehsan, Malaysia). Subsequently, DNA bands were photographed using G-box gel documentation (Syngene, USA).

### Statistical analysis

Statistical analyses were performed in GraphPad Prism8 (San Diego, CA, USA). Statistical values of all experimental results were calculated using an analysis of variance (ANOVA) followed by Dunnett’s test. Differences were compared at confidence levels of *P* < 0.05, *P* < 0.01 and *P* < 0.001. All data are expressed as the mean ± SD carried out at least in triplicate.

### Supplementary Information

Supplementary Information.

## Data Availability

All data generated or analyzed during this study are included in this published article and its supplementary information files.
