# Supplementary material for: Rational design and characterization of cell-selective antimicrobial peptides based on a bioactive peptide from Crocodylus siamensis hemoglobin
Source: Sci Rep. 2023 Sep 26;13:16096. doi: 10.1038/s41598-023-43274-9 (PMC10522709; doi:10.1038/s41598-023-43274-9)
Supplement: Supplementary file 1 — Supplementary Information. [file 41598_2023_43274_MOESM1_ESM.docx]

**Supplementary information**

**Rational design and characterization of cell-selective antimicrobial peptides based on a bioactive peptide from *Crocodylus siamensis* hemoglobin**

**Sirinthip Sosiangdi^1,2^, Lapatrada Taemaitree^3^, Anupong Tankrathok^1,4^, Sakda Daduang^1,5^, Sophon Boonlue^1,6^, Sompong Klaynongsruang^1,2^ & Nisachon Jangpromma^1,2*^**

^1^*Protein and Proteomics Research Center for Commercial and Industrial Purposes (ProCCI), Faculty of Science, Khon Kaen University, Khon Kaen 40002, Thailand*

^2^*Department of Biochemistry, Faculty of Science, Khon Kaen University, Khon Kaen 40002, Thailand*

^3^*Department of Integrated Science, Faculty of Science, Khon Kaen University, Khon Kaen 40002, Thailand*

^4^*Department of Biotechnology, Faculty of Agricultural Technology, Kalasin University, Kalasin 46000, Thailand*

^5^*Department of Pharmacognosy and Toxicology, Faculty of Pharmaceutical Sciences, Khon Kaen University, Khon Kaen, 40002, Thailand*

^6^*Department of Microbiology, Faculty of Science, Khon Kaen University, Khon Kaen 40002, Thailand*

***Corresponding author**

Email address: nisaja@kku.ac.th (N. Jangpromma)

**Table S1.** A table of the concentration that causes 50% hemolysis (HC_50_) and the concentration corresponding to 50% cell death (IC_50_) by the peptides

| **Peptide**  **name** | **Hemolysis concentration_50_ (HC_50_) (μg/mL)** | **IC_50_ (μg/mL)** | |
| --- | --- | --- | --- |
|  |  | **PBMC** | **HaCaT** |
| QL17 | >2000 | >200 | >200 |
| IL15 | >2000 | >200 | >200 |
| IL15.1 | >2000 | >200 | >200 |
| IL15.2 | >2000 | >200 | >200 |
| IL15.3 | >2000 | >200 | >200 |
| IL15.4 | >2000 | >200 | >200 |
| IL15.5 | 896 | 113 | 200 |

**Table S2.** Selectivity index of the peptides against Gram-negative bacteria.

| Peptide  name | Selectivity index (SI) | | | | | | | | | | |
| --- | --- | --- | --- | --- | --- | --- | --- | --- | --- | --- | --- |
|  | ***E. coli*** | | |  | ***K. pneumonia*** | | |  | ***P. aeruginosa*** | | |
|  | **Erythrocytes** | **PBMC** | **HaCaT** |  | **Erythrocytes** | **PBMC** | **HaCaT** |  | **Erythrocytes** | **PBMC** | **HaCaT** |
| QL17 | - | - | - |  | - | - | - |  | - | - | - |
| IL15 | - | - | - |  | - | - | - |  | - | - | - |
| IL15.1 | - | - | - |  | - | - | - |  | - | - | - |
| IL15.2 | 20 | 2 | 2 |  | - | - | - |  | - | - | - |
| IL15.3 | 500 | 50 | 50 |  | 200 | 20 | 20 |  | 200 | 20 | 20 |
| IL15.4 | 500 | 50 | 50 |  | 143 | 14 | 14 |  | 200 | 20 | 20 |
| IL15.5 | 60 | 8 | 13 |  | 12 | 2 | 3 |  | 26 | 3 | 6 |

**Table S3.** Selectivity index of the peptides against Gram-positive bacteria.

| Peptide  name | Selectivity index (SI) | | | | | | | | | | |
| --- | --- | --- | --- | --- | --- | --- | --- | --- | --- | --- | --- |
|  | ***S. aureus*** | | |  | ***B. subtilis*** | | |  | ***S. epidermidis*** | | |
|  | **Erythrocytes** | **PBMC** | **HaCaT** |  | **Erythrocytes** | **PBMC** | **HaCaT** |  | **Erythrocytes** | **PBMC** | **HaCaT** |
| QL17 | - | - | - |  | - | - | - |  | - | - | - |
| IL15 | - | - | - |  | - | - | - |  | - | - | - |
| IL15.1 | - | - | - |  | - | - | - |  | - | - | - |
| IL15.2 | - | - | - |  | 40 | 4 | 4 |  | - | - | - |
| IL15.3 | 167 | 17 | 17 |  | 250 | 25 | 25 |  | 222 | 22 | 22 |
| IL15.4 | 167 | 17 | 17 |  | 333 | 33 | 33 |  | 222 | 22 | 22 |
| IL15.5 | 36 | 5 | 8 |  | 112 | 14 | 25 |  | 75 | 9 | 17 |


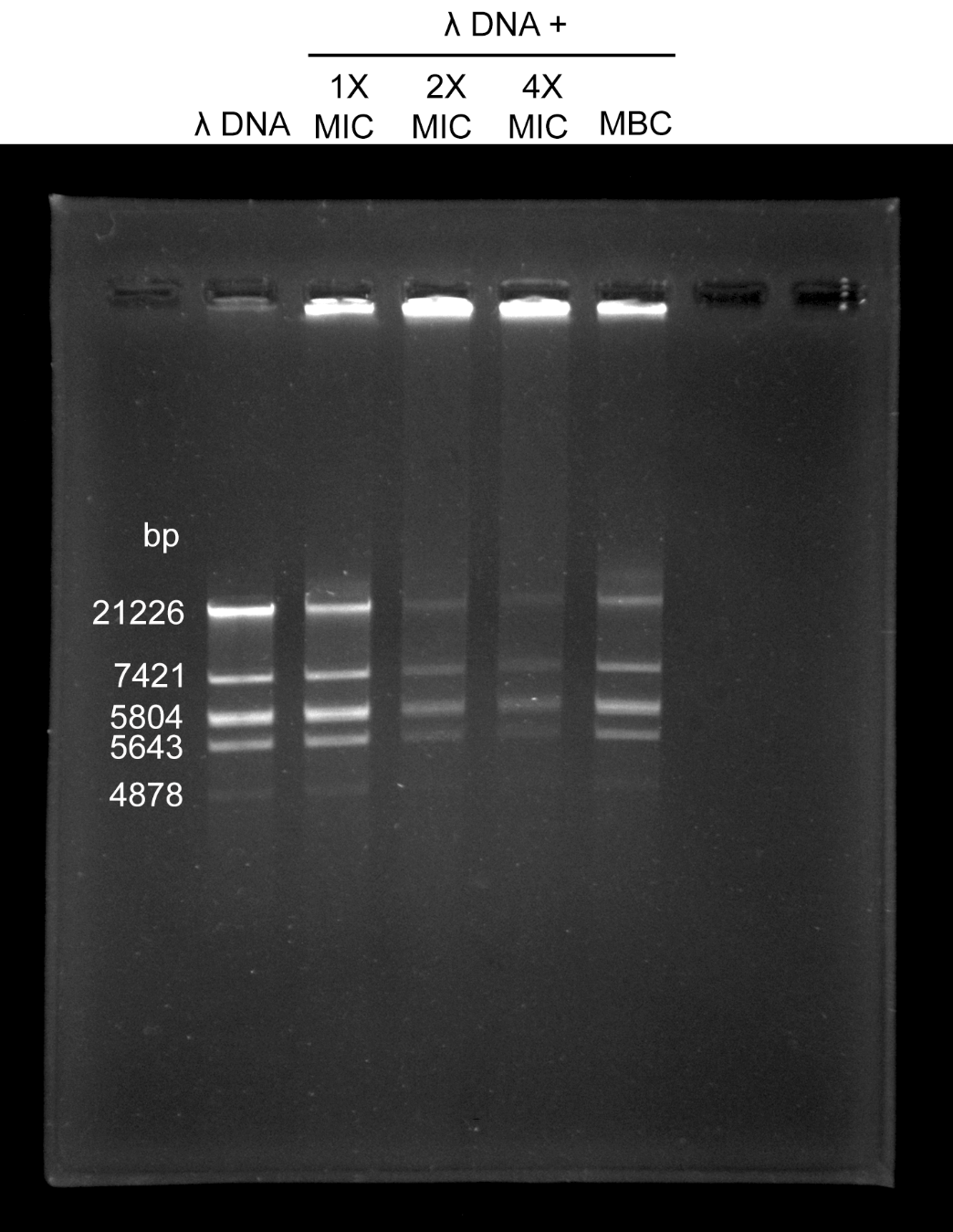


**Figure S1.** Concentration-dependent binding of IL15.3 peptide with DNA as shown by a gel retardation assay and smearing of the DNA bands at higher concentrations of the peptide. EcoRI-digested Lambda (λ) DNA (200 ng) was incubated with different concentrations of IL15.3 at 1🞩MIC (4 µg/mL), 2🞩MIC (8 µg/mL), 4🞩MIC (16 µg/mL) and MBC (6 µg/mL) of *E. coli*. The results were analyzed using 0.8 % agarose gel electrophoresis.
